# End-Stage Kidney Failure in Oman: An Analysis of Registry Data with an Emphasis on Congenital and Inherited Renal Diseases

**DOI:** 10.1155/2017/6403985

**Published:** 2017-06-08

**Authors:** Intisar Al Alawi, Issa Al Salmi, Adhra Al Mawali, Yacoub Al Maimani, John A. Sayer

**Affiliations:** ^1^National Genetic Centre, Royal Hospital, Muscat, Oman; ^2^Institute of Genetic Medicine, Newcastle University, Central Parkway, Newcastle upon Tyne NE1 3BZ, UK; ^3^The Renal Medicine Department, Royal Hospital, Muscat, Oman; ^4^Centre of Studies and Research, Ministry of Health, Muscat, Oman; ^5^The Renal Dialysis Centre, Royal Hospital, Muscat, Oman

## Abstract

Globally, end-stage kidney disease (ESKD) is a huge burden on health care systems. The aims of this study were to perform a comprehensive epidemiological and etiological report of ESKD patients commencing RRT in Oman with an emphasis on genetic causes and inherited kidney disease. All newly registered Omani patients with ESKD commencing RRT from 2001 until 2015 (*n* = 2,922) were analysed using the RRT register in Oman. All potentially genetic or inherited causes of ESKD were reviewed. In Oman, ESKD is more prevalent in males (57.1%) than females (42.9%) with a median age of incident ESKD of 53 years. Diabetic nephropathy was the most prevalent cause of ESKD (46%), followed by hypertensive nephropathy (19%), glomerulonephritis (15%), and inherited kidney disease (5%). For patients less than 20 years of age inherited kidney disease accounted for 32.5% of cases. Of this cohort with inherited renal disease, 40.3% had autosomal dominant polycystic kidney disease, 11.5% had congenital anomalies of the kidney and urinary tract, 9.4% had Alport syndrome, and 7.2% had autosomal recessive polycystic kidney disease. This study represents a comprehensive population-based epidemiological and etiological report of ESKD patients in Oman commencing RRT. Inherited kidney disease was the leading cause of paediatric ESKD.

## 1. Introduction

Chronic kidney disease (CKD) is a common condition characterized by irreversible kidney damage and reduced glomerular filtration rates that may progress to end-stage kidney disease (ESKD), where renal replacement therapy (RRT) is necessary for long term survival. The number of patients receiving RRT worldwide in 2010 was estimated to be 2.6 million, whereas the estimated number of actual patients demanding RRT was 4.9 million [1]. This RRT gap is one of the global challenges presented with the growing rate of ESKD.

Inherited kidney diseases are important causes of morbidity and may lead to both progressive CKD and ESKD. Inherited kidney disease accounts for approximately 20% of all CKD cases and is an important cause of ESKD [2]. Renal registry studies suggest that at least 10% of ESKD in adults is related to inherited renal disease, with autosomal dominant polycystic kidney disease (ADPKD) making a large proportion of these cases [3]. In the United States (US) and Europe, congenital anomalies of the kidney and urinary tract (CAKUT) and inherited nephropathies are the major causes of CKD among youngest ESKD groups [4]. The situation in the Middle East countries was revealed to be the same, but the prevalence of inherited kidney disease is reported to be much higher (up to 30%) compared to Europe due to high rates of consanguinity [4].

Oman is the second largest country in the South East of Arabian Peninsula (309,500 square kilometres) with a relatively small and young population (3,831,553 people). In Oman, there has been a progressive increase in the ESKD incidence and prevalence over the last three decades [5]. The incidence rate of ESKD patients receiving RRT in Oman at the end of 1998 was 21 per million population (PMP), whereas the calculated incidence in 2013 was 120 PMP [5]. Along with the increasing rate of ESKD, a gradual increase in morbidity caused by CKD had been observed.

Ministry of Health data from Oman reports that 39% of perinatal deaths in hospitals are associated with congenital malformations and genetic disorders [6] and the consanguinity rates were found to be higher (73%) between parents of newborns with major congenital malformations [7]. The prevalence of some rare inherited kidney diseases such as cystinuria and autosomal recessive polycystic kidney disease (ARPKD) was reported to be higher compared to the worldwide prevalence due to high consanguinity [8, 9]. However, there is no comprehensive data estimating the magnitude of inherited kidney disease in patients in Oman. Here we review ESKD patients commencing RRT in Oman over a fifteen-year period and report the proportion and clinical characteristics of congenital and inherited kidney disease.

## 2. Materials and Methods

### 2.1. Ethical Approvals

This study is part of an original research titled “Genetic Studies of Inherited Kidney Disease in Oman,” which was ethically approved by the Research and Ethical Review and Approval Committee, Ministry of Health (MH/DGP/R&S/PROPOSAL_APPROVED/18/2014), and by the Ethics Committee, College of Medicine and Health Sciences, Sultan Qaboos University (MREC #1096).

### 2.2. Retrospective Analysis

Data from newly registered Omani patients with ESKD commencing RRT from 2001 to 2015 was analysed using renal replacement therapy register in Oman. The registry contains baseline characteristics of patients at initiation of RRT and updates until patient death. The baseline characteristic information included is sex, comorbid conditions (including diabetes mellitus, hypertension, ischemic heart disease, cerebrovascular disease, and respiratory disease), family history of disease, initial hypertension medications, and initial pre-RRT BMI, serum albumin, and creatinine. Haemodialysis data, peritoneal dialysis data, and renal transplantation data were also included in the registry. This data is completed by nephrologists in all renal dialysis units throughout the country once the patient reached ESKD using a standardized form and sent to the main renal dialysis unit in Muscat, where the RRT registry is maintained in a comprehensive database [5]. The registry data collection in Oman has been standardized as much as possible to allow meaningful comparisons to other countries. The data set collected is similar to that collected by the USRDS (form 2728) [5].

All potentially congenital, genetic, or hereditary causes of kidney diseases were extracted from the registry within the study period according to the coding protocol of this registry. Cases registered and classified under the code Hereditary Familial Renal Disease (HFRD) were reviewed. This code includes the majority of inherited kidney diseases including ADPKD, ARPKD, Alport syndrome, primary hyperoxaluria, cystic dysplastic kidney, nephronophthisis, Bartter syndrome, and inherited renal tubular acidoses. The proportion of inherited kidney disease among those commencing RRT was calculated. Statistical analysis was performed using IBM SPSS Statistics 20.

## 3. Results and Discussion

### 3.1. Characteristics of ESKD Patients Commencing RRT in Oman

**Figure 1:**
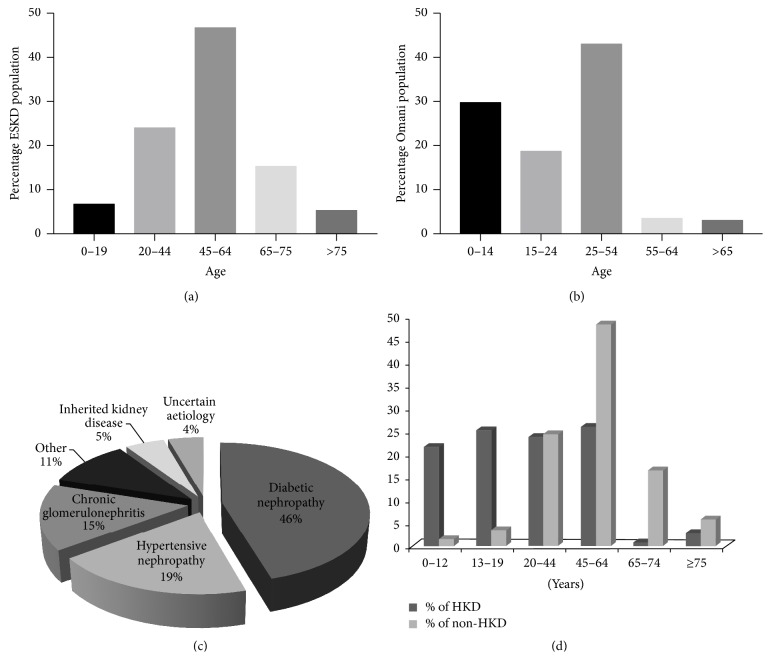
*Characteristics of ESKD patients commencing RRT in Oman*. (a) Percentage age distribution of patients with ESKD. (b) Percentage age distribution of Omani population (2014 data). (c) Aetiology of ESKD in Oman. (d) Comparison of percentage of patients with hereditary kidney disease (HKD) and nonhereditary kidney disease (non-HKD) across age groups.

From 2001 to 2015, a total of 2,922 new patients commenced RRT due to different causes. Males contributed 57.1% (*n* = 1668) of the patients and females contributed 42.9% (*n* = 1254). The mean age of RRT commencement was 50.14 ± SD 17.5 years, while the median age was 53 years. Overall, 1321 (47.1%) cases of ESKD occurred among patients who were 45–64 years, whereas 884 (31.5%) occurred among patients who were ≤44 years and 599 (21.2%) in patients who were 65 years and over (Figure 1(a)).

Diabetic nephropathy was the most prevalent cause of ESKD (46%), followed by hypertensive nephropathy (HTN) (19%) and chronic glomerulonephritis (CGN) (15%) (Figure 1(c)). Inherited kidney disease contributed just 5% of the total RRT population. Other aetiologies, such as urological, tubulointerstitial kidney disease and vascular causes, comprised 11% of RRT population. However, a dramatically different picture was revealed when the primary diagnosis is given by age groups (Tables 1 and 2). In patients less than 20 years of age, inherited kidney disease was the most common primary cause of kidney disease (Table 1) accounting for 42% of ESRD in 0–12 years' age group and 27% of ESRD in the 13–19 years' age group (Table 2). Therefore, in patients less than 20 years of age inherited kidney disease accounted for 62 of 200 cases (32.5%) of ESRD. A comparison of Omani registry data from comparator countries shows a much smaller burden of ADPKD in Oman (just 2%) than Western countries (Table 3). The reasons for this probably reflect the relative young population in Oman. An age comparison of the total population of Omani versus age of ESKD population confirms this (Figures 1(a) and 1(b)). Published data from Australia and New Zealand describe an increase in the contribution of ADPKD to ESRD over the last 5 decades [15] and reflect an overall increase in age of the ESRD population. Similar observations have been made in other countries including Denmark [16] and the USA [17]. Thus, over the next few decades the relative contribution of ADPKD to ESRD in Oman will likely increase towards 5–10%.

### 3.2. Characteristics of Inherited Kidney Disease Patients on ESKD

The distribution of males with inherited kidney disease was found to be higher (*n* = 79; 56.8%) than females (*n* = 60; 43.2%). Patients with inherited kidney disease started RRT at a younger age with a mean of 29.4 ± SD 20.1 and median age of 21 years (Figure 1(d)). A positive family history of inherited kidney disease was present in 36.7% of inherited kidney disease cases. Dialysis was the initial RRT modality in all inherited kidney disease patients (*n* = 115) except 24 patients, who received preemptive transplant in the form of living-related donor (*n* = 6) and living nonrelated donor (*n* = 18) (Table 4). Currently, in the cohort of inherited kidney disease patients, 37% have a functioning transplant, 38% are receiving haemodialysis, and 3.6% are receiving peritoneal dialysis (Table 4). A total of 25 patients (18%) have died during the study period with cardiac disease being the leading cause of death. Hypertension was the most common comorbidity in inherited kidney disease patients at initiation of RRT (54.7%), compared to diabetes (2.2%), ischemic heart disease (3.6%), cerebrovascular diseases (1.4%), and respiratory diseases (1.4%) (Table 4). ADPKD was the most common inherited kidney disease diagnosis, accounting for 40.3% of cases, followed by CAKUT (11.5%), Alport syndrome (9.4%), and ARPKD (7.2%) (Table 5).

This study represents a comprehensive, up-to-date population-based epidemiological and etiological report of Oman patients reaching ESKD and commencing RRT. It reveals that ESKD is more prevalent in males, with a ratio of 1.3, which is consistent with data reported from other countries [18]. The median age of incident ESKD patients starting RRT was 53 years and is in agreement with that reported in other Middle East countries with almost similar demographics and socioeconomic features, including Saudi Arabia [19] and Jordan [10], but substantially lower than that reported in European countries, including United Kingdom (UK) [11], Croatia, Georgia and Cyprus [20], and the US [21]. The data we present here from Oman shows that there is a sharp increase in the prevalence of ESKD with increasing age. We anticipate that as the population ages in Oman, the ESKD prevalence will increase. This trend is consistent with that reported from Saudi Arabia, which is comparable to the situation in developed countries where the rate of elderly is recently decreased or stabilized [19].

Diabetic nephropathy was the commonest underlying cause of ESKD and was more prevalent among older patients of 45 years and over. Eighteen years ago, the prevalence of diabetic nephropathy among RRT patients in Oman was reported to be 14.5% [22], whereas in our study it accounted for 45.5% of ESKD population. The observed incidence of diabetic nephropathy leading to ESKD places Oman among the highest countries in the world. Table 3 allows a comparison in the percentage of incident ESKD patients due to diabetes mellitus in Oman and other countries.

In this study, we described for the first time the prevalence of inherited kidney disease causing ESKD in Oman. Inherited kidney disease comprises 5% of all causes of ESKD but was highly prevalent in paediatric ESKD patients. The detected prevalence of hereditary kidney disease in our study is considerably lower than that reported in other countries including Libya (12%) [10] and Australia (10%) [23]; however, it is consistent with the summarized estimate of the countries of the Gulf Cooperation Council (GCC) of 4.43% [24]. Nonetheless, inherited and congenital kidney disease is thought to be an important aetiology of ESKD in Omani population in which the consanguinity rate is relatively high (56.3%) [25]. The detected low frequencies of inherited kidney disease are expected to be due to high mortality rate among newborns with recessively inherited kidney disorders. CKD in paediatric patients is a devastating illness and the mortality rate for those with ESKD receiving RRT is expected to be 30–150 times higher when compared to a general paediatric population [26]. Moreover, since what is seen from ESKD is only the “tip of the iceberg” of CKD we expect inherited kidney disease patients with earlier stages of CKD are probably exceeding those reaching ESRD. At present, there are no means to capture this important data.

As expected, ADPKD was the most prevalent inherited kidney disease, accounting for 2% of total ESKD population. This prevalence is substantially lower than that reported in developed countries including the UK (9.9%) [11] and lower than the summarized estimate of ADPKD in GCC countries (4.8%), likely reflecting a younger ESKD population in Oman [24].

## 4. Conclusions

In summary, this study represents a population-based etiological report of Omani ESKD commencing RRT from 2001 to 2015. It clearly shows that Oman is facing major factors that globally are fundamentally responsible for the growing incidence of ESKD in adults, namely, an aging population and a high burden of diabetes mellitus. Therefore, health care providers must concentrate on strategic actions that highlight primary prevention, early detection, and dynamic management of CKD population. For the first time, the prevalence of inherited kidney disease causing ESKD in Oman has been accurately described and this data emphasizes need to measure the frequencies of inherited kidney disease patients in earlier stages of CKD and assess their rate of progression to ESKD.

## Figures and Tables

**Table 1:** Distribution of primary kidney diagnosis by age in ESKD patients (2001–2015).

Primary causes of end-stage kidney disease														
Age group (years)	Diabetic nephropathy		Hypertensive nephropathy		Glomerulonephritis		Inherited kidney disease		Other		Uncertain aetiology		Total	
	*N*	%	*N*	%	*N*	%	*N*	%	*N*	%	*N*	%	*N*	%
0–12	2	0.1	0	0	16	0.6	30	1.1	15	0.5	8	0.3	71	2.5
13–19	0	0	4	0.1	45	1.6	35	1.2	33	1.2	12	0.4	129	4.6
20–44	173	6.2	125	4.5	187	6.7	33	1.2	119	4.2	47	1.7	684	24.4
45–64	769	27	253	9	139	5	36	1.3	77	2.7	47	1.7	1321	47.1
65–74	259	9.2	110	3.9	27	1	1	0	36	1.3	7	0.2	440	15.7
≥75	73	2.6	49	1.7	10	0.4	4	0.1	19	0.7	4	0.1	159	5.7

Total	1276	*45.5%*	541	*19.3%*	424	*15.1%*	139	*5%*	299	*10.6%*	125	*4.5%*	*2804*	*100%*

Percentages calculated after excluding patients without primary diagnosis data.

**Table 2:** Distribution of inherited kidney disease by age in ESKD patients (2001–2015).

Age group (years)	Inherited kidney disease		Remaining causes		Total
	*N*	%	*N*	%	*N*
0–12	30	42.3	41	57.7	71
13–19	35	27.1	94	72.9	129
20–44	33	4.8	651	95.2	684
45–64	36	2.7	1285	97.3	1321
65–74	1	0.2	439	99.8	440
≥75	4	2.5	155	97.5	159

Total	139	*5*	2665	*95*	*2804*

**Table 3:** Primary causes of ESKD in Oman and other countries.

Country	Diabetic	Hypertension	Glomerulonephritis	Uncertain aetiology	ADPKD	Reference
Jordan	29.2	14.2	12.3	12.4	—	[10]
United Kingdom	15.9	6.1	19	16	9.9	[11]
Libya	26.5	14.6	21.2	10.2	6.3	[12]
India	20.5	4.5	34.5	19	5	[13]
Pakistan	10	12	37	19	3	[13]
Turkey	29.9	25.9	7.9	15.7	3.8	[14]
*Oman*	*45.5*	*19.3*	*15.1*	*4.5*	*2*	*This study*

**Table 4:** Comparison between ADPKD and other causes of hereditary kidney disease.

	ADPKD		Other inherited kidney diseases		Total inherited kidney disease population	
	*N*	%	*N*	%	*N*	%
*Positive family history of disease*	23	41	28	33.7	51	37
*Comorbidity*						
Diabetes mellitus	2	3.6	1	1.2	3	2.2
Hypertension	34	61	42	50.6	76	55
Ischemic heart disease	5	8.9	0	0	5	3.6
Cerebrovascular disease	1	1.8	1	1.2	2	1.4
Respiratory disease	1	1.8	1	1.2	2	1.4
Other	6	11	17	20.5	23	17
*First RRT modality*						0
Haemodialysis	42	75	63	75.9	105	76
Peritoneal dialysis	1	1.8	9	10.8	10	7.2
preemptive transplant, living related donor	1	1.8	5	6.02	6	4.3
preemptive transplant, living nonrelated donor	12	21	6	7.23	18	13
*Current status*						
Haemodialysis	18	32	35	40.7	53	38
Peritoneal dialysis	1	1.8	4	4.65	5	3.6
Transplant	22	39	30	34.9	52	37
Lost to follow-up	2	3.6	1	1.16	3	2.2
Deceased	13	23	12	14	25	18
*Cause of death*						
Cardiac disease	5	38	3	25	8	32
Cerebrovascular disease	2	15	1	8.33	3	12
Infection	3	23	2	16.7	5	20
Other	1	7.7	4	33.3	5	20
Uncertain	2	15	2	16.7	4	16

**Table 5:** Inherited kidney diseases in ESKD population (2001–2015).

Inherited kidney disease	Number	Proportion of inherited kidney disease (%)	Proportion of ESRD in this cohort (%)	Age group (*N*)			
				0–12	13–19	20–44	45+
Autosomal dominant polycystic kidney disease (ADPKD)	56	40.3	2	1	1	17	37
Congenital anomalies of kidney and urinary tract (CAKUT)	16	11.5	0.6	8	6	2	0
Alport syndrome	13	9.4	0.5	0	7	6	0
Autosomal recessive polycystic kidney disease (ARPKD)	10	7.2	0.4	3	6	0	1
Dysplastic cystic kidney	7	5	0.2	1	3	2	1
Steroid resistant nephrotic syndrome (congenital & childhood)	5	3.6	0.2	4	1	0	0
Primary hyperoxaluria	5	3.6	0.2	2	2	1	0
Prune-belly syndrome	5	3.6	0.2	2	2	1	0
Familial focal segmental glomerulosclerosis	5	3.6	0.2	1	2	2	0
Medullary cystic kidney	4	2.9	0.1	3	0	1	0
Familial interstitial nephropathy	2	1.4	0.1	1	1	0	0
Haemolytic uremic syndrome	2	1.4	0.1	2	0	0	0
Mesangioproliferative glomerulosclerosis	2	1.4	0.1	0	1	1	0
Membranoproliferative glomerulosclerosis	2	1.4	0.1	0	0	0	2
Nephronophthisis	1	0.7	0	1	0	0	0
Bartter syndrome	1	0.7	0	0	1	0	0
Lowe's syndrome	1	0.7	0	0	1	0	0
Renal tubular acidosis	1	0.7	0	0	1	0	0
Undetermined familial disease	1	0.7	0	1	0	0	0

Total	*139*	*100%*	*5%*	*30*	*35*	*33*	*36*
